# Not all moderate disease is the same – Identification of disability trajectories among patients with rheumatoid arthritis and moderate disease activity

**DOI:** 10.1371/journal.pone.0215999

**Published:** 2019-05-20

**Authors:** Yi Pan, Sam Norton, James M. Gwinnutt, Lianne Kearsley-Fleet, Deborah P. M. Symmons, Mark Lunt, Adam Young, BSRBR-RA Control Centre Consortium, Kimme L. Hyrich, Suzanne M. M. Verstappen

**Affiliations:** 1 Arthritis Research UK Centre for Epidemiology, Centre for Musculoskeletal Research, Division of Musculoskeletal and Dermatological Sciences, School of Biological Sciences, Faculty of Biology, Medicine and Health, University of Manchester, Manchester Academic Health Science Centre, Manchester, United Kingdom; 2 Health Psychology section, Institute of Psychiatry, Psychology and Neuroscience, King’s College London, London, United Kingdom; 3 Department of Inflammation Biology, Faculty of Life Sciences and Medicine, King’s College London, London, United Kingdom; 4 Centre for Health Services & Clinical Research & Post Graduate Medicine, University of Hertfordshire, College Lane, Hatfield, United Kingdom; 5 NIHR Manchester Biomedical Research Centre, Manchester University Hospitals NHS Foundation Trust, Manchester Academic Health Science Centre, Manchester, United Kingdom; Universita Campus Bio-Medico di Roma, ITALY

## Abstract

**Background:**

United Kingdom guidelines for the use of biologic disease modifying anti-rheumatic drugs (bDMARDS) for rheumatoid arthritis (RA) require patients to have active disease (Disease Activity Score [DAS28] >5.1) and have failed ≥2 previous conventional synthetic DMARDs (csDMARD). Patients with moderate disease activity (MDA) do not meet these criteria, yet often have poor outcomes. This study aimed to identify trajectory groups of disability scores over three years in RA patients with MDA.

**Methods:**

The study included biologic-naïve patients receiving csDMARDs only with MDA (3.2 <DAS28≤ 5.1) when recruited to the control cohort of the British Society for Rheumatology Biologics Register–RA (BSRBR-RA). Disability scores, measured using the Health Assessment Questionnaire (HAQ), were recorded every six months for three years. Trajectories of HAQ scores over follow-up were assessed using latent class growth models (LCGMs). Baseline age, gender, DAS28, symptom duration, rheumatoid factor status, number of prior csDMARDs and co-morbidities were assessed as potential predictors of group membership.

**Results:**

In total, 1274 patients were included (mean age: 61 years (standard deviation: 12), 71.4% women). The best fitting model included seven HAQ trajectories. These trajectories were horizontal over follow-up and were related to baseline HAQ: very-low (6.8%, baseline (BL) HAQ: 0.22), low (11.5%, BL HAQ: 0.41), low-moderate (17.0%, BL HAQ: 0.93), moderate (13.4%, BL HAQ: 1.09), high-moderate (19.5%, BL HAQ: 1.61), severe (23.2%, BL HAQ: 1.98) and very-severe (8.6%, BL HAQ: 2.54). Higher DAS28, older age, female gender, longer disease duration and more co-morbidities were independently associated with higher HAQ trajectory group.

**Conclusion:**

There is substantial heterogeneity in baseline HAQ scores in this population, and the trajectories of HAQ scores after baseline are, on average, relatively flat. As bDMARD therapy has been shown to improve HAQ scores, patients with MDA but high HAQ scores may benefit from a more aggressive approach to therapy.

## Introduction

Rheumatoid arthritis (RA) is a chronic autoimmune condition, which is associated with inflammation of synovial joints and may result in increased disability and reduced quality of life [1]. Over the past two decades, the treatment of RA has been revolutionised by the introduction of biologic disease modifying anti-rheumatic drugs (bDMARDs) [2]. These drugs have been demonstrated to be effective at lowering disease activity, improving functional ability, and reducing mortality [3,4]. However, the strict United Kingdom (UK) National Institute for Health and Care Excellence (NICE) guidelines require patients to have high disease activity (Disease Activity Score 28 [DAS28] >5.1), and have failed two conventional synthetic DMARDs (csDMARDs) [5], before starting a bDMARD.

Patients with moderate disease activity (MDA; 3.2 < DAS28 ≤ 5.1) and who thus fail to reach the criteria for bDMARD treatment in the UK, have poor long-term outcomes [6–8]. For example, patients from the French *Etude et Suivi des Polyarthrites Indifférenciées Récentes* (ESPOIR) cohort assessed between the six and 12 month visits who had persistent MDA had two-fold increased odds of radiographic damage and higher disability scores (measured using the Heath Assessment Questionnaire (HAQ)) at three years compared with patients in remission [6]. Similarly, patients from the UK Early Rheumatoid Arthritis Network (ERAN) cohort with DAS28 <3.2 at year one had seven-fold increased odds of having low DAS28 and threefold increased odds of having low HAQ scores at year two compared with patients with MDA at year one [7]. In another UK study, almost a quarter of patients (21.4%) with persistent MDA recruited to the Yorkshire Early Arthritis Register had increases in HAQ score above the minimum clinically important difference over a six month period [8]. Furthermore, research has shown that the administration of bDMARDs to patients with MDA leads to improved outcomes compared with methotrexate alone [9,10].

Previous studies assessing patients with MDA have analysed the cohorts as single homogenous groups. However, as the DAS28 definition for MDA is wide (1.9 units) and as the DAS28 is made up of 4 separate components, this group is likely to be heterogeneous. The total MDA population may be made up of subgroups characterised by distinct trajectories of long-term outcome such as functional ability. Therefore, the aim of this study was to determine whether there are multiple trajectories of HAQ scores over three years in patients with MDA at baseline, and to identify predictors of group membership where they exist. This research will allow the identification of groups of patients with MDA who are likely to have worse long-term outcomes and who may benefit from more aggressive therapy.

## Patients and methods

### Study population

The British Society for Rheumatology Biologics Register for RA (BSRBR-RA) is an ongoing national, observational cohort study of outcomes among patients receiving bDMARDs for RA [11]. In order to place any observed adverse events into context and to understand the risks of bDMARDs compared to csDMARDs, a cohort of 3800 patients with RA and at least MDA (but not eligible for biologic therapy), was also recruited in parallel between 2002 and 2009 [12]. The BSRBR-RA csDMARD group included patients with early RA starting csDMARDs for the first time and patients with more longstanding RA who had received more than one csDMARD prior to recruitment. Patients from the BSRBR-RA csDMARD cohort were included in this analysis if they had MDA at study registration (baseline) (3.2 < DAS28 ≤ 5.1) and had HAQ recorded at baseline (N = 1274). Ethical approval was granted by the North West Multicentre Research Ethics Committee in December 2000 (MREC 00/8/53) and patients gave their written informed consent to participate and for their data to be used in further analyses investigating the long-term outcomes of RA.

### Data collection

At study registration, data were extracted from patients and from medical records and included age, gender, symptom duration, baseline HAQ score, baseline DAS28, rheumatoid factor (RF) status, information about prior csDMARD use and co-morbidities, which were selected from a pre-determined list of conditions. Outcome data, including changes in treatment, disease activity, HAQ scores and adverse events were collected every six months for three years.

### Statistical analysis

Descriptive statistics were used to summarise the baseline characteristics of the patients. Distinct trajectories of functional disability (HAQ, bounded between 0 and 3) over time were identified using latent class growth models (LCGMs) [13]. The model included two parts: (a) a censored normal based trajectory model using time polynomials as covariates, such as linear, quadratic and cubic terms of years since registration; (b) a multinomial logistic regression model for class membership using baseline characteristics as predictors. Predictors assessed were: baseline age, gender, symptom duration, DAS28, RF positivity, number of prior csDMARDs and number of co-morbidities (composite variable generated from the presence of high blood pressure, ischaemic heart disease, stroke, lung disease, renal disease, liver disease, diabetes mellitus or depression).

Patients with missing baseline HAQ data were excluded, but patients with some missing follow-up HAQ scores were included and contributed data to other time-points within the model. The LCGM analysis was performed using the Stata plugin traj [14]. In order to determine the number of latent classes (trajectory groups), initially a single class of LCGM was fitted which is equivalent to a simple linear regression model and then models allowing up to nine latent groups were estimated. Both Akaike's information criterion (AIC) and (adjusted) Bayesian information criterion (adjusted BIC) were used to select the optimum model, with values closest to zero indicating better model fit. Since the second part of the model employs multinomial logistic regression, we imposed a further stipulation that no latent class could contain less than 5% of the total sample (n<64) for reasons of power and precision. After determining the best-fitting model, we then examined the association of these groups with demographic and clinical baseline characteristics using multinomial logistic regression analysis. Cox proportional hazards models were then used to assess the association between trajectory group membership and subsequent death and starting a biologic over the first three years of follow-up. Patients were censored after three years.

Missing data on baseline predictors were low (14 missing data-points out of 8918 [0.2%]). Multiple imputation using iterative chained equations was used to account for these missing data, with 10 imputed datasets created. Imputed variables were only used as predictors in the multinomial logistic regression model. A sensitivity analysis was conducted, limiting the analysis to patients with ≤2 years symptom duration at baseline (N = 395, 31%). All analyses were performed using Stata 13.1 (StataCorp. 2013. Stata Statistical Software: Release 13. College Station, TX: StataCorp LP).

## Results

A total of 1274 csDMARD patients had MDA at study registration and completed baseline HAQ and were therefore included. These patients had a mean age (standard deviation) at baseline of 61.1 (12.3) years and 910 (71.4%) were women (Table 1). Of those included in the analysis, 63 (5.0%) patients died during follow-up and 49 (3.9%) patients switched to bDMARD therapy within the first three years following registration.

**Table 1:** Baseline characteristics of the analysis cohort, mean (standard deviation) displayed unless otherwise stated.

	% missing	BSRBR-RA, N = 1274	Min	Max
Age at registration, years	0	61.1 (12.3)	18	90
Gender, N (%) female	0	910 (71.4)		
Symptom duration, years	1.0	10.3 (10.6)	0	65
HAQ score	0	1.36 (0.75)	0	3
DAS28 score	0	4.34 (0.50)	3.20	5.10
Rheumatoid factor, N (%) positive	0.08	755 (59.3)		
Number of prior DMARDs	0	2.4 (1.7)	0	10
Co-morbidity, n (%)	0			
None		516 (40.5)		
1 co-morbidity		447 (35.1)		
2 co-morbidities		216 (17.0)		
≥ 3 co-morbidities		95 (7.5)		

DAS28 = Disease Activity Score (28), DMARD = disease modifying anti-rheumatic drug, HAQ = health assessment questionnaire, N = number

Co-morbidities included: high blood pressure, ischaemic heart disease, stroke, lung disease, renal disease, liver disease, diabetes mellitus, depression

**Fig 1:**
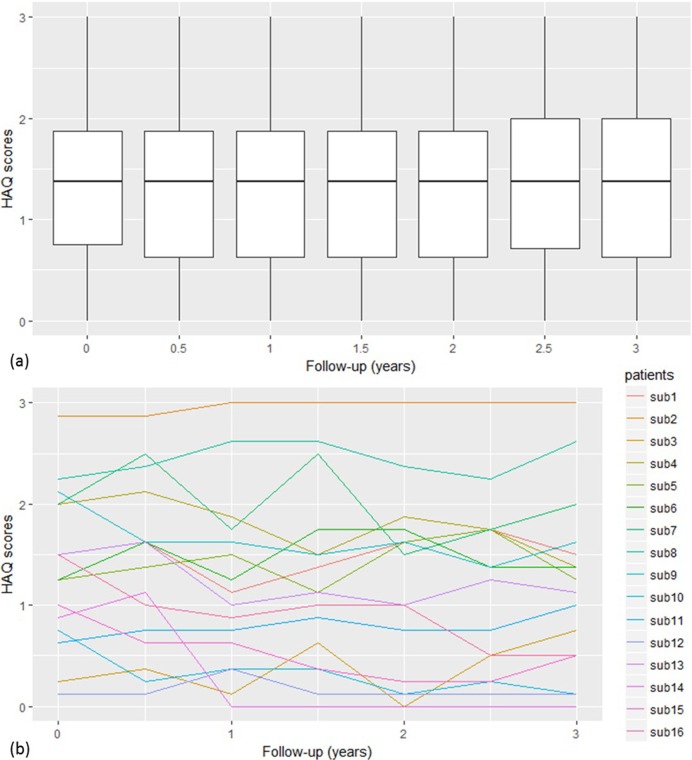
Box plot of HAQ scores distribution and HAQ trajectories for 16 random subjects.

The median number of HAQ scores per patient was 6 (IQR 3, 7) and the median HAQ score at each assessment over the course of three years was 1.38 (IQR 0.63, 1.88). At the sample level, the progression of HAQ score was stable over time (Fig 1A). However, sample level statistics, such as the median, can mask the between-individual variability in HAQ score over time as shown by the trajectories of 16 randomly selected patients (Fig 1B). Therefore, LCGMs were fitted, allowing for several distinct HAQ trajectories over time.

### Latent class growth models

#### Trajectory groups

**Fig 2:**
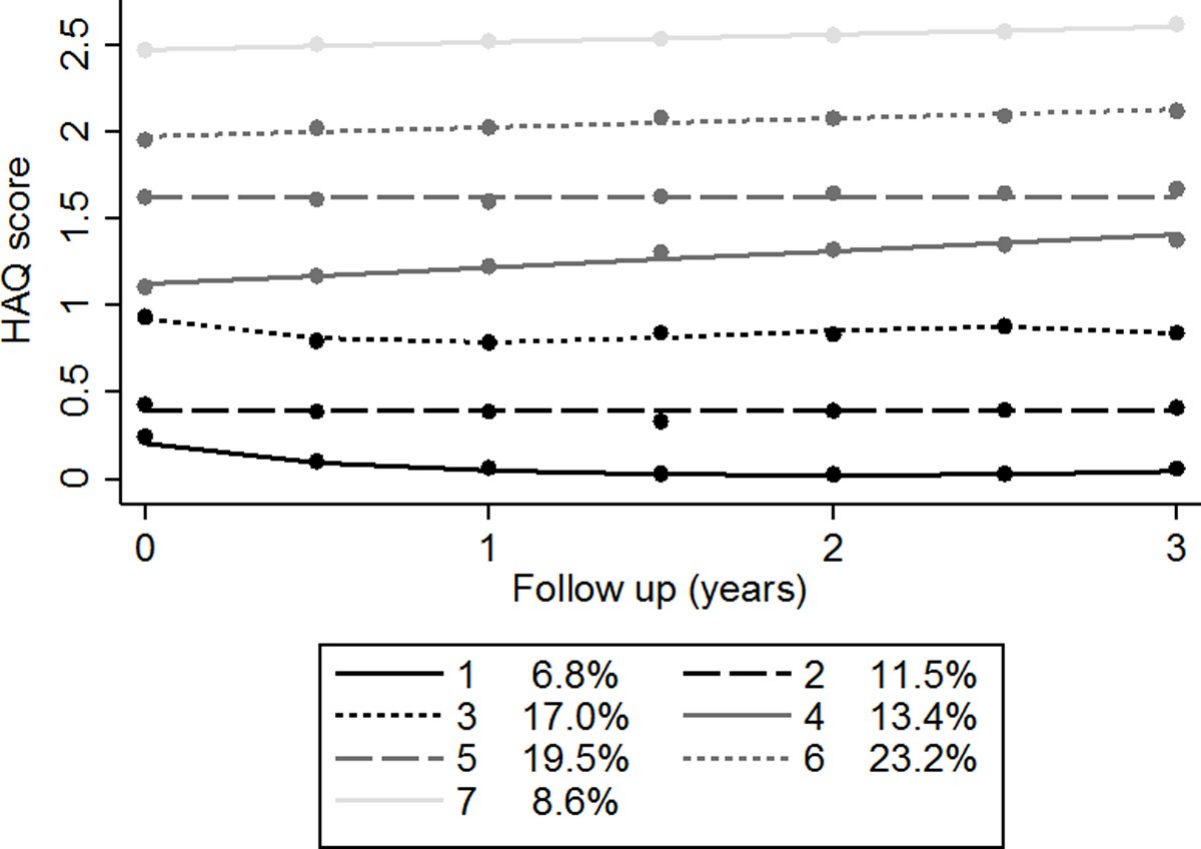
HAQ disability trajectories for the 7 latent classes.

The fit of models with one to nine latent classes were evaluated. A model including seven latent classes was selected as having the best (i.e. most parsimonious) fit to the data. There was heterogeneity in the HAQ scores of patients with MDA at baseline (Fig 2). The HAQ trajectories of patients from baseline were horizontal and were associated with the HAQ score of each trajectory at baseline. The groups were labelled as follows, based on baseline HAQ: very-low (Group 1, 6.8%, mean baseline (BL) HAQ: 0.22), low (Group 2, 11.5%, BL HAQ: 0.41), low-moderate (Group 3, 17.0%, BL HAQ: 0.93), moderate (Group 4, 13.4%, BL HAQ: 1.09), high-moderate (Group 5, 19.5%, BL HAQ: 1.61), severe (Group 6, 23.2%, BL HAQ: 1.98) and very-severe (Group 7, 8.6%, BL HAQ: 2.54).

#### Trajectory group characteristics

The baseline characteristics of the seven trajectory groups are summarized in Table 2. There were clear correlations between increasing trajectory group (i.e. worsening of HAQ scores) and older age, higher proportion of females, longer disease duration, worse baseline HAQ score, worse baseline DAS28, higher proportion of RF+ patients, more prior csDMARDs used and more co-morbidities. When assessing baseline predictors of HAQ score using multinomial logistic regression, the strongest independent predictor of group membership was DAS28 (relative risk ratio per unit increase in baseline DAS28 [95% CI]: trajectory 1 = 1 [reference category], trajectory 2 = 0.94 [0.55, 1.58]; trajectory 3 = 1.71 [1.04, 2.82], trajectory 4 = 2.12 [1.25, 3.60], trajectory 5 = 2.31 [1.40, 3.83], trajectory 6 = 2.57 [1.56, 4.25], trajectory 7 = 3.14 [1.70, 5.81]). Other baseline factors that showed an independent association with trajectory group were age, gender, disease duration, number of prior csDMARDs and number of co-morbidities, whereas positive RF was not associated with higher trajectory group (Table 3). There was no significant relationship between increasing HAQ trajectory group and death during follow-up or starting a biologic (see S1 Table). The sensitivity analysis produced similar results to the main analyses.

**Table 2:** Baseline characteristics of the analysis cohort by trajectory group (n = 1274), mean (standard deviation) displayed unless otherwise stated.

Trajectory Group	1	2	3	4	5	6	7
	Very-Low	Low	Low-moderate	Moderate	High-moderate	Severe	Very-severe
Number of patients, n (%)	87 (6.8)	146 (11.5)	217 (17.0)	171 (13.4)	248 (19.5)	296 (23.2)	109 (8.6)
Age at registration, years	57.5 (12.3)	56.9 (11.7)	57.4 (12.7)	63.0 (11.7)	62.1 (12.0)	63.1 (12.1)	65.8 (10.6)
Gender, n (%) female	55 (63.2)	91 (62.3)	160 (73.7)	112 (65.5)	178 (71.8)	220 (74.3)	94 (86.2)
Symptom duration, years	6.0 (7.3)	7.0 (8.2)	7.8 (8.8)	9.6 (10.4)	10.8 (10.9)	12.1 (10.5)	18.7 (13.4)
HAQ	0.22 (0.33)	0.41 (0.33)	0.93 (0.37)	1.09 (0.36)	1.61 (0.31)	1.98 (0.31)	2.54 (0.23)
DAS28	4.21 (0.52)	4.17 (0.52)	4.33 (0.51)	4.36 (0.47)	4.38 (0.50)	4.40 (0.48)	4.44 (0.44)
RF+, n (%)	51 (58.6)	80 (54.8)	118 (54.4)	97 (56.7)	147 (59.3)	186 (63.1)	76 (69.7)
Prior DMARDs	1.7 (1.4)	2.0 (1.5)	2.0 (1.6)	2.2 (1.7)	2.4 (1.5)	2.8 (1.7)	3.2 (1.7)
No co-morbidity	55 (63.2)	79 (54.1)	124 (57.1)	66 (38.6)	86 (34.7)	74 (25.0)	32 (29.4)
1 co-morbidity	20 (23.0)	41 (28.1)	70 (32.3)	68 (39.8)	96 (38.7)	112 (37.8)	40 (36.7)
2 co-morbidities	11 (12.6)	20 (13.7)	14 (6.5)	27 (15.8)	46 (18.6)	75 (25.3)	23 (21.1)
≥3 co-morbidities	1 (1.2)	6 (4.1)	9 (4.2)	10 (5.9)	20 (8.1)	35 (11.8)	14 (12.8)

DAS28 = Disease Activity Score (28), DMARD = disease modifying anti-rheumatic drug, HAQ = Health Assessment Questionnaire, N = number, RF = rheumatoid factor

**Table 3:** Baseline independent predictors of trajectory group membership, results are relative risk ratios (95% confidence intervals) with trajectory group 1 as the reference category.

Trajectory Group	1	2	3	4	5	6	7
	Very-Low	Low	Low-moderate	Moderate	High-moderate	Severe	Very-severe
Age at registration	1(ref)	0.99 (0.97, 1.01)	1.00 (0.98, 1.02)	1.03 (1.00, 1.05)	1.02 (1.00, 1.04)	1.02 (1.00, 1.04)	1.04 (1.01, 1.07)
Female vs male	1(ref)	0.93(0.53, 1.62)	1.52(0.89, 2.62)	1.10(0.63, 1.92)	1.45(0.85, 2.49)	1.71(1.00, 2.94)	3.84(1.83, 8.03)
Symptom duration, years	1(ref)	1.01(0.97, 1.05)	1.02(0.99, 1.06)	1.03(0.99, 1.07)	1.04(1.00, 1.08)	1.04(1.01, 1.08)	1.08(1.04, 1.12)
DAS28	1(ref)	0.94(0.55, 1.58)	1.71(1.04, 2.82)	2.12(1.25, 3.60)	2.31(1.40, 3.83)	2.57(1.56, 4.25)	3.14(1.70, 5.81)
RF+ vs RF-	1(ref)	0.80(0.46, 1.33)	0.76(0.45, 1.27)	0.78(0.45, 1.33)	0.84(0.50, 1.41)	0.94(0.56, 1.58)	1.14(0.60, 2.13)
Prior DMARDs	1(ref)	1.24(0.97, 1.57)	1.16(0.92, 1.46)	1.30(1.02, 1.64)	1.35(1.06, 1.68)	1.54(1.24, 1.92)	1.57(1.23, 1.99)
1 co-morbidity vs no co-morbidity	1(ref)	1.47(0.77, 2.80)	1.57(0.86, 2.87)	2.54(1.36, 4.77)	2.88(1.57, 5.28)	3.99(2.16, 7.36)	3.28(1.57, 6.82)
2 co-morbidities vs no co-morbidity	1(ref)	1.33(0.58, 3.04)	0.59(0.25, 1.40)	1.79(0.80, 4.01)	2.49(1.16, 5.34)	4.76(2.23, 10.13)	3.29(1.34, 8.04)
≥3 co-morbidities vs no co-morbidity	1(ref)	4.53(0.52, 39.30)	4.54(0.55, 37.37)	7.00(0.85, 57.60)	11.98(1.53, 94.01)	24.16(3.12, 187.1)	21.58 (2.58, 180.2)

DAS28 = Disease Activity Score (28), DMARD = Disease modifying Anti-Rheumatoid Drug, N = number, RF = rheumatoid factor

## Discussion

This study aimed to describe the heterogeneity in disability over three years of RA patients with baseline MDA by identifying distinct trajectories of HAQ score. There was significant heterogeneity in HAQ scores at baseline within this patient group, despite all having moderate disease activity. A seven trajectory group model fitted the follow-up data best, but each of these trajectories was characterised by relatively constant HAQ scores, associated with baseline HAQ. This is in spite of csDMARD therapy, indicating the potential for more aggressive treatment strategies in patients with MDA and high HAQ scores.

Currently, the treatment decision to start a patient with RA on a bDMARD is driven solely by DAS28. To select patients early who could potentially benefit from treatment with a biologic, it may be important to consider DAS28 and HAQ score together, given the results of this paper indicating significant heterogeneity in HAQ scores in a cohort of patients with MDA. The NICE cost-effectiveness model for biologics explicitly included variability in baseline HAQ score into their models [5], and a working group aiming to achieve consensus in decision models for biologics in RA concluded HAQ should be included in economic models [15], indicating the potential importance of thinking about HAQ and DAS28 when selecting patients to start biologic therapy.

LCGMs have previously been applied to the HAQ scores of patients with inflammatory arthritis included in two UK inception cohorts (the Early Rheumatoid Arthritis Study [ERAS], and the Norfolk Arthritis Register [NOAR]) [16,17]. These analyses reported four HAQ trajectory groups, as opposed to the seven reported in this analysis. This could be due to differences in entry criteria for the different studies (e.g. this analysis: prevalent cases with MDA; ERAS, NOAR analyses: inception cases with any disease activity) or due to differences in follow-up length (this study: 3 years; ERAS: 10 & 15 years; NOAR: 15 years) or differences in the criteria used to determine the optimal number of trajectory classes (this study: AIC and BIC; ERAS: Likelihood ratio test; NOAR: validation of ERAS groups). The analyses of ERAS and NOAR also assessed predictors of group membership, reporting that baseline older age, female sex, longer symptom duration and higher DAS28 were associated with higher HAQ trajectory group membership, in line with the results from this analysis [17]. However, while we found no association with mortality, in ERAS where mortality was considered, individuals in a high stable and moderate increasing trajectory group were observed to be associated with increased mortality risk [16]. However, there were relatively few deaths across the seven trajectories in the current analysis, meaning that the survival analysis in this study may be underpowered.

More comorbidity was also associated with higher HAQ trajectory group in the current analysis. Patients with conditions such as fibromyalgia and other musculoskeletal conditions alongside their RA are likely to be over-represented in the higher HAQ trajectories as well as higher disease activity groups [18,19]. However, as data on fibromyalgia were not collected consistently in the BSRBR-RA, we cannot test this.

The current study has a number of strengths. The large sample size and the multiple repeated assessments allow the construction of robust LCGMs. A wide range of demographic and clinical predictors were available to be screened as predictors of group membership. Patients came from 28 different centres across the UK, meaning the results can be generalised to patients across the country. A weakness of this research is that there were some missing baseline HAQ scores for some patients, and hence these patients were not included in the analysis. However, the number of patients excluded was low (N = 137) and the baseline characteristics of these patients did not differ from patients with measured baseline HAQ score and therefore any bias due to complete-case analysis is likely to be minimal. Furthermore, HAQ score is a subjective measure of functional disability and there were no objective measures of function within the BSRBR to corroborate the heterogeneity observed in the HAQ scores. A further weakness is that no other disease activity measures were collected within the BSRBR-RA, (e.g. the Clinical Disease Activity Index or the Simplified Disease Activity Index) meaning we could not assess the heterogeneity of HAQ scores in a cohort of moderate disease activity patients defined using a disease activity measure other than the DAS28.

In conclusion, this analysis has identified seven distinct HAQ trajectory groups in patients who have MDA at baseline, indicating that this is a heterogeneous population with a range of potential long-term outcomes. These trajectories were relatively stable over time, despite csDMARD therapy. Biologic therapies have been shown to improve physical function in RA patients with MDA [9,10,20]. This study indicates that some patients with RA and MDA have high levels of disability and these patients are likely to continue having high disability over subsequent years. These patients should be identified and may benefit from more aggressive therapy, such as biologic therapy, despite their moderate disease activity.

### BSRBR-RA Control Centre Consortium

The BSRBR-RA Control Centre Consortium recruited a cohort of patients receiving conventional synthetic disease-modifying anti-rheumatic drugs (csDMARDs) between 2002 and 2009. This Consortium consists of the following institutions (all in the UK): Antrim Area Hospital, Antrim (Dr Nicola Maiden), Cannock Chase Hospital, Cannock Chase (Dr Tom Price), Christchurch Hospital, Christchurch (Dr Neil Hopkinson), Royal Derby Hospital, Derby (Dr Sheila O’Reilly), Dewsbury and District Hospital, Dewsbury (Dr Lesley Hordon), Freeman Hospital, Newcastle-upon-Tyne (Dr Ian Griffiths), Gartnavel General Hospital, Glasgow (Dr Duncan Porter), Glasgow Royal Infirmary, Glasgow (Prof Hilary Capell), Haywood Hospital, Stoke-on-Trent (Dr Andy Hassell), Hope Hospital, Salford (Dr Romela Benitha), King's College Hospital, London (Dr Ernest Choy), Kings Mill Centre, Sutton-In Ashfield (Dr David Walsh), Leeds General Infirmary, Leeds (Prof Paul Emery), Macclesfield District General Hospital, Macclesfield (Dr Susan Knight), Manchester Royal Infirmary, Manchester (Prof Ian Bruce, Prof Kimme Hyrich), Musgrave Park Hospital, Belfast (Dr Allister Taggart), Norfolk and Norwich University Hospital, Norwich (Prof David Scott), Poole General Hospital, Poole (Dr Paul Thompson), Queen Alexandra Hospital, Portsmouth (Dr Fiona McCrae), Royal Glamorgan Hospital, Glamorgan (Dr Rhian Goodfellow), Russells Hall Hospital, Dudley (Prof George Kitas), Selly Oak Hospital, Selly Oak (Dr Ronald Jubb), St Helens Hospital, St Helens (Dr Rikki Abernethy), Weston General Hospital, Weston-super-Mare (Dr Shane Clarke/Dr Sandra Green), Withington Hospital, Manchester (Dr Paul Sanders), Withybush General Hospital, Haverfordwest (Dr Amanda Coulson), North Manchester General Hospital (Dr Bev Harrison), Royal Lancaster Infirmary (Dr Marwan Bukhari) and The Royal Oldham Hospital (Dr Peter Klimiuk).

## Supporting information

S1 Table Outcomes over follow-up, stratified by trajectory group. † Hazard ratios calculated using Cox regression adjusted for age and gender.
‡ Group 2 chosen as reference category for biologic switching analysis, as group 1 only had one failure event, and this resulted in failures of the proportional hazards assumption. The proportional hazards assumption was met when group 2 was used as the reference category.
CI = confidence interval
bDMARD = biologic disease modifying anti-rheumatic drug.
(DOCX)

## Abbreviations

AIC: Akaike’s Information criterion
bDMARD: biologic disease modifying anti-rheumatic drugs
BIC: Bayesian Information Criterion
BL: baseline
BSRBR-RA: British Society for Rheumatology Biologics Register for RA
CI: confidence interval
csDMARD: conventional synthetic disease modifying anti-rheumatic drug
DAS28: Disease Activity Score (28 joints)
ERAN: Early Rheumatoid Arthritis Network
ERAS: Early Rheumatoid Arthritis Study
ESPOIR: Etude et Suivi des Polyarthrites Indifférenciées Récentes
HAQ: Health Assessment Questionnaire
IQR: interquartile range
LCGM: latent class growth model
MDA: moderate disease activity
NICE: National Institute for Health and Care Excellence
NOAR: Norfolk Arthritis Register
RA: rheumatoid arthritis
RF: rheumatoid factor
UK: United Kingdom
