# Correction: Not all moderate disease is the same – Identification of disability trajectories among patients with rheumatoid arthritis and moderate disease activity

**DOI:** 10.1371/journal.pone.0231481

**Published:** 2020-04-02

**Authors:** Yi Pan, Sam Norton, James M. Gwinnutt, Lianne Kearsley-Fleet, Deborah P. M. Symmons, Mark Lunt, Adam Young, BSRBR-RA Control Centre Consortium, Kimme L. Hyrich, Suzanne M. M. Verstappen

In the ‘Trajectory group characteristics’ subsection of the Results, there is an error in the fourth sentence. The correct sentence is: There was no significant relationship between increasing HAQ trajectory group and death during follow-up, but some higher trajectory groups had greater proportion of patients who started a biologic (see S1 Table).

There are several errors in S1 Table. Please view the correct S1 Table below.

## Supporting information

S1 Table Outcomes over follow-up, stratified by trajectory group. † Hazard ratios calculated using Cox regression adjusted for age and gender.
‡ Group 2 chosen as reference category for biologic switching analysis, as group 1 only had one failure event, and this resulted in failures of the proportional hazards assumption. The proportional hazards assumption was met when group 2 was used as the reference category.
CI = confidence interval
bDMARD = biologic disease modifying anti-rheumatic drug.
(DOCX)
