# Supplementary material for: Not all moderate disease is the same – Identification of disability trajectories among patients with rheumatoid arthritis and moderate disease activity
Source: PLoS One. 2019 May 20;14(5):e0215999. doi: 10.1371/journal.pone.0215999 (PMC6527224; doi:10.1371/journal.pone.0215999)
Supplement: S1 Table — † Hazard ratios calculated using Cox regression adjusted for age and gender. ‡ Group 2 chosen as reference category for biologic switching analysis, as group 1 only had one failure event, and this resulted in failures of the proportional hazards assumption. The proportional hazards assumption was met when group 2 was used as the reference category. CI = confidence interval bDMARD = biologic disease modifying anti-rheumatic drug. (DOCX) [file pone.0215999.s001.docx]

*Supplementary table 1 – Outcomes over follow-up, stratified by trajectory group*

*† Hazard ratios calculated using Cox regression adjusted for age and gender*

*‡ Group 2 chosen as reference category for biologic switching analysis, as group 1 only had one failure event, and this resulted in failures of the proportional hazards assumption. The proportional hazards assumption was met when group 2 was used as the reference category.*

*CI = confidence interval*

*bDMARD = biologic disease modifying anti-rheumatic drug*

| Trajectory Group | 1 | 2 | 3 | 4 | 5 | 6 | 7 |
| --- | --- | --- | --- | --- | --- | --- | --- |
|  | **Low Decreasing** | **Low Stable** | **Moderate Stable** | **Moderate Increasing** | **High Stable** | **High Increasing** | **Severe Increasing** |
| Died during follow-up,  n (%) | 5 (5.8) | 1 (0.7) | 6 (2.8) | 9 (5.3) | 15 (6.1) | 19 (6.4) | 8 (7.3) |
| Hazard ratio of death,  (95% CI) † | 1  (ref) | 0.11  (0.01, 0.97) | 0.51  (0.16, 1.69) | 0.61  (0.20, 1.84) | 0.79  (0.28, 2.18) | 0.81  (0.30, 2.20) | 0.95  (0.30, 2.97) |
| Switched to bDMARD during follow-up, n (%) | 1 (1.2) | 4 (2.7) | 5 (2.3) | 6 (3.5) | 14 (5.7) | 15 (5.1) | 4 (3.7) |
| Hazard ratio for switching to biologic, (95% CI) † | 0.42  (0.05, 3.77) | 1  (ref ‡) | 0.85  (0.23, 3.16) | 1.67  (0.47, 5.96) | 2.69  (0.88, 8.23) | 2.50  (0.82, 7.57) | 2.15  (0.53, 8.81) |
