# Supplementary material for: Correction: Not all moderate disease is the same – Identification of disability trajectories among patients with rheumatoid arthritis and moderate disease activity
Source: PLoS One. 2020 Apr 2;15(4):e0231481. doi: 10.1371/journal.pone.0231481 (PMC7117688; doi:10.1371/journal.pone.0231481)
Supplement: S1 Table — † Hazard ratios calculated using Cox regression adjusted for age and gender. ‡ Group 2 chosen as reference category for biologic switching analysis, as group 1 only had one failure event, and this resulted in failures of the proportional hazards assumption. The proportional hazards assumption was met when group 2 was used as the reference category. CI = confidence interval bDMARD = biologic disease modifying anti-rheumatic drug. (DOCX) [file pone.0231481.s001.docx]

**S1 Table**. **Outcomes over follow-up, stratified by trajectory group.**

| Trajectory Group | 1 | 2 | 3 | 4 | 5 | 6 | 7 |
| --- | --- | --- | --- | --- | --- | --- | --- |
|  | **Very low** | **Low** | **Low-moderate** | **Moderate** | **High-moderate** | **Severe** | **Very-severe** |
| Died during follow-up,  n (%) | 5 (5.8) | 1 (0.7) | 6 (2.8) | 9 (5.3) | 15 (6.1) | 19 (6.4) | 8 (7.3) |
| Hazard ratio of death,  (95% CI) † | 1  (ref) | 0.11  (0.01, 0.97) | 0.51  (0.16, 1.69) | 0.61  (0.20, 1.84) | 0.79  (0.28, 2.18) | 0.81  (0.30, 2.20) | 0.95  (0.30, 2.97) |
| Switched to bDMARD during follow-up, n (%) | 2 (2.3) | 11 (7.5) | 19 (8.8) | 20 (11.7) | 32 (12.9) | 44 (14.9) | 6 (5.5) |
| Hazard ratio for switching to bDMARD, (95% CI) † | 0.30  (0.07, 1.36) | 1  (ref ‡) | 1.19  (0.57, 2.50) | 2.04  (0.97, 4.27) | 2.29  (1.15, 4.56) | 2.75  (1.41, 5.36) | 1.13  (0.41, 3.08) |

*† Hazard ratios calculated using Cox regression adjusted for age and gender*

*‡ Group 2 chosen as reference category for biologic switching analysis, as group 1 had a small number of events, and this resulted in wide confidence intervals.*

*CI = confidence interval, bDMARD = biologic disease modifying anti-rheumatic drug*
